# Immobilizing calcium-dependent affinity ligand onto iron oxide nanoparticles for mild magnetic mAb separation

**DOI:** 10.1016/j.btre.2024.e00864

**Published:** 2024-11-26

**Authors:** Ines Zimmermann, Friederike Eilts, Anna-Sophia Galler, Jonas Bayer, Sophia Hober, Sonja Berensmeier

**Affiliations:** a Chair of Bioseparation Engineering, TUM School of Engineering and Design, Technical University of Munich, Boltzmannstraße 15, 85748 Garching, Germany; b Munich Institute of Integrated Materials, Energy and Process Engineering, Technical University of Munich, Lichtenbergstraße 4a, 85748 Garching, Germany; c Department of Protein Science, KTH Royal Institute of Technology, 10691 Stockholm, Sweden

**Keywords:** Anything but conventional chromatography (abc), Downstream processing, Epoxy, Silica, Physical and covalent immobilization

## Abstract

Current downstream processing of monoclonal antibodies (mAbs) is limited in throughput and requires harsh pH conditions for mAb elution from Protein A affinity ligands. The use of an engineered calcium-dependent ligand (Z_Ca_) in magnetic separation applications promises improvements due to mild elution conditions, fast processability, and process integration prospects. In this work, we synthesized and evaluated three magnetic nanoparticle types immobilized with the cysteine-tagged ligand Z_Ca_-cys. Ligand homodimers were physically immobilized onto bare iron oxide nanoparticles (MNP) and MNP coated with tetraethyl orthosilicate (MNP@TEOS). In contrast, Z_Ca_-cys was covalently and more site-directedly immobilized onto MNP coated with (3-glycidyloxypropyl)trimethoxysilane (MNP@GPTMS) via a preferential cysteine-mediated epoxy ring opening reaction. Both coated MNP showed suitable characteristics, with MNP@TEOS@Z_Ca_-cys demonstrating larger immunoglobulin G (IgG) capacity (196 mg *g*^−1^) and the GPTMS-coated particles showing faster magnetic attraction and higher IgG recovery (88 %). The particles pave the way for the development of calcium-dependent magnetic separation processes.

## Highlights

• Calcium-dependent magnetic mAb separation aims to enhance downstream processing.
• Affinity ligand Z_Ca_-cys was immobilized onto three iron oxide nanoparticle types.
• Physical immobilization enabled higher but less oriented loadings than covalent.
• High mAb binding capacities were reached (up to 196 mg *g^-1^*).
• High mAb recoveries were achieved at pH 5.5 (up to 88 %).

## Graphical abstract

**Figure:**
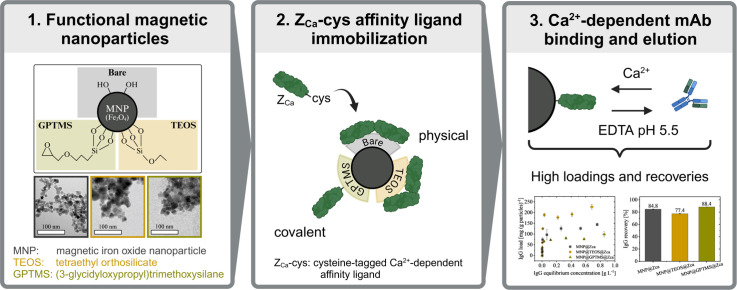

## 1 Introduction

Monoclonal antibodies (mAbs) are essential in the treatment of several diseases. The market for therapeutic mAbs and derived biosimilar products is constantly growing, accompanied by increasing drug approvals [[1], [2], [3]]. For efficient mAb production, research has focused on optimizing expression systems and engineering the upstream processing (USP). This resulted in highly increased expression productivity over the last decades [4,5]. Currently, the main bottleneck of manufacturing lies in the subsequent mAb purification, the downstream processing (DSP), which is based on a central Protein A affinity chromatography capture step [6,7]. Despite excellent yields and purities achieved with state-of-the-art Protein A chromatography [8,9], essential drawbacks are the need for acidic elution conditions to desorb mAbs from Protein A ligands [10,11] and the limited throughput due to mass transport limitations in the packed column bed [8,12].

Acidic elution can cause unwanted conformational changes in mAb products, resulting in product degradation, aggregation, and loss of functionality [10,[13], [14], [15]]. The low pH elution has been the subject of several research studies [[16], [17], [18], [19]]. These studies focused on milder elution conditions while preserving the selective affinity between Protein A and mAbs. In a promising approach, Kanje et al. developed an engineered, calcium-dependent Protein A-based ligand (Z_Ca_) [19]. The Z_Ca_ ligand is based on a Z-domain derived from the B-domain of native Protein A. A calcium-binding loop grafted between helices two and three of the three-helix molecule allows calcium-dependent antibody binding. In the presence of calcium, mAbs bind to the ligand. In contrast, the ligand undergoes conformational changes and releases the mAbs upon calcium depletion, e.g., by ethylenediaminetetraacetic acid (EDTA), sodium chloride (NaCl), or citrate buffers [19,20]. Thus, mAbs can be eluted at mild pH values between 5.5 and 7 [20].

So far, the Z_Ca_ ligand has only been applied in packed-bed chromatography [[19], [20], [21], [22]], a method restricted by mass transfer limitations. Thus, we aim to implement the promising calcium-dependent ligand in magnetic separation applications. Magnetic separation is based on magnetically controllable adsorbents, usually functional magnetic particles that are freely dispersed in the analyte solution. Together with other researchers [[23], [24], [25], [26], [27]], we believe magnetic separation can enhance mAb DSP as an alternative to conventional packed-bed chromatography. Our group focuses on using magnetic iron oxide nanoparticles as they show several advantages for separation processes. Firstly, nanoparticles have a large specific surface area accessible for interactions with target molecules [28]. Secondly, the particles are generally non-porous, which significantly reduces mass transfer limitations compared to porous chromatography resins and thus benefits fast kinetics and high throughput. In a previous study on magnetic mAb separation, we demonstrated 90 % mAb binding within 30 s [29]. Thirdly, the non-porosity allows for the potential processing of unclarified cell culture broth from USP because clogging of the separation matrix is prevented [6,23,24]. This step integration reduces the number of process steps and thereby process time and costs, thus offering the prospect of process intensification. Lastly, no column-packing is required for the application, and the adsorption process is theoretically independent of the scale, simplifying scale-ups [30].

**Fig. 1:**
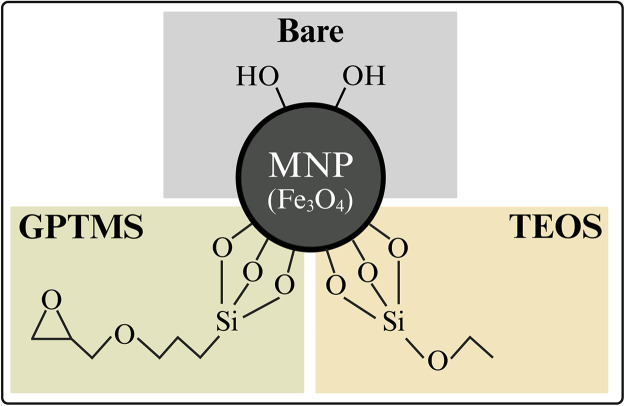
Schematic illustration of the three particle types investigated for immobilizing the cysteine-tagged, calcium-dependent Protein A ligand (Z_Ca_). MNP: magnetic iron oxide nanoparticle, TEOS: tetraethyl orthosilicate, GPTMS: (3-glycidyloxypropyl)trimethoxysilane. Note that the hydrolyzed silane coupling agents (TEOS and GPTMS) react with hydroxyl groups (-OH) of bare MNP by condensation. However, a high degree of crosslinking between the multiple silanol groups also occurs (forming Si-O-Si bonds), which is, for simplicity, not shown here.

In the present study, we took the first step towards combining the mentioned advantages of the calcium-dependent affinity ligand Z_Ca_ and the magnetic separation technique. Aiming to develop functional magnetic particles, we synthesized three nanoparticle types (Fig. 1) and investigated physical and covalent immobilization strategies of the cysteine (cys)-tagged Z_Ca_ ligand. Physical immobilization strategies onto magnetic nanoparticles based on, e.g., electrostatic interactions, hydrophobic interactions, hydrogen bonds, or coordinative bonding are generally chosen due to their simplicity [27,31] and their negligible impact on magnetic particle characteristics [29]. On the other hand, covalent immobilization usually requires specific particle coatings but can enable more robust and specific immobilization [32]. In our study, we analyzed the physical ligand immobilization directly onto bare iron oxide magnetic nanoparticles (MNP), intending to preserve the particle characteristics of MNP that are beneficial for magnetic separation processes, e.g., large specific surface areas and high magnetizations [29]. In addition, MNP are produced by a simple, low-cost particle synthesis. In a second approach, we examined the physical Z_Ca_-cys immobilization onto MNP coated with tetraethyl orthosilicate (TEOS) as a reference to the bare particles. TEOS is frequently employed in functional particle coatings and was used for physical peptide immobilization before [[33], [34], [35]]. Lastly, we studied MNP coated with (3-glycidyloxypropyl)trimethoxysilane (GPTMS) because of their simple and cheap one-step MNP modification and their potential for cys-mediated, covalent protein binding by nucleophilic epoxy ring opening [[36], [37], [38]].

The three synthesized particle types were first characterized using Fourier-transform infrared (FT-IR) spectroscopy and transmission electron microscopy (TEM) to ensure successful particle synthesis. Additional analyses for the comparative evaluation of the particles included the determination of specific surface areas from nitrogen sorption isotherms, the assessment of zeta potentials, the investigation of agglomeration in process buffers through dynamic light scattering (DLS), and the evaluation of magnetic properties using a superconducting quantum interference device (SQUID). Afterward, we focused on the Z_Ca_-cys immobilization onto the three particles, investigating the maximum ligand capacity, the immobilization stability, and the impact of the cys-tag. For the latter, we compared the immobilization of two additional recombinant affinity ligands with and without cys-tag (rSpA and rSpA-cys). Finally, we examined the IgG adsorption in a Ca^2+^-containing buffer and the subsequent desorption in EDTA (pH 5.5) to prove the functionality of the immobilized ligands. Our work reveals interesting insights into different ligand immobilization strategies and is a proof-of-concept of calcium-dependent magnetic separation of mAbs.

## 2 Materials and methods

### 2.1 Magnetic nanoparticle synthesis

Bare magnetic iron oxide nanoparticles (MNP) were synthesized by co-precipitation following the previously reported procedure [29]. MNP were the basis for the synthesis of the coated particles.

#### 2.1.1 Tetraethyl orthosilicate (TEOS) functionalization

The TEOS coating was synthesized according to the method reported earlier by our group [35]. Bare MNP (0.92 g) were stabilized in 180 mL citric acid (60 mM) using ultrasonication (5 min, 20 % amplitude, 10 s on/ 15 s off; UP400 St; Hielscher, Germany). After titration to neutral pH using 25 % tetraethylammonium hydroxide, the particles were added into a 4 L round bottom flask with 2.72 L ethanol, 180 mL ammonium hydroxide, 720 mL degassed water, and 1.74 mL TEOS. The suspension was sonicated (ultrasonic bath USC – TH; VWR, Germany) for 1 h at 4 °C under a nitrogen atmosphere. After the reaction, the MNP@TEOS were washed with ethanol and bi-distilled, degassed water, and stored at 4 °C under nitrogen until further use.

#### 2.1.2 (3-Glycidyloxypropyl)trimethoxysilane (GPTMS) functionalization

For GPTMS functionalization, the method reported by Ghaemy et al. was modified [39]. In short, 120 mg bare MNP were suspended in 3.6 mL ethanol/bi-distilled water (v/v 50/50) and treated in an ultrasonic bath (USC – TH; VWR, Germany) for 30 min. In a 25 mL round bottom flask, the particle suspension was stirred with 500 µL GPTMS for 4 h at 80 °C under nitrogen, followed by 12 h at room temperature. After several washing steps with ethanol and bi-distilled water, the MNP@GPTMS were stored under nitrogen at 4 °C.

### 2.2 Magnetic nanoparticle characterization

All measurements for particle characterization were performed at room temperature, if not stated differently. Particle concentrations were determined using dry weight measurements and a phenanthroline assay as described previously [29].

Particles were visually analyzed by transmission electron microscopy (TEM) using a TEM JEM 1400 Plus device (Jeol, Japan). For preparation, 0.025 g L^−1^ of particle suspension in water was sonicated until homogenous (ultrasonic bath USC – TH; VWR, Germany). Afterward, 30 µL of the suspension was pipetted onto a carbon grid sample holder and dried with heated air. The mean particle size of MNP was determined based on several microscopic images by measuring the size (longest dimension) of one hundred particles using ImageJ software (National Institutes of Health, USA).

Attenuated total reflection (ATR) Fourier-transform infrared (FT-IR) spectra were generated using an Alpha II device (Bruker Corporation, USA) to analyze functional groups. Per sample, 24 scans were measured over a wave number range of 4000–400 cm^−1^. Before the measurement, all samples (1 g L^−1^) were rebuffered into deionized water. Recorded spectra were edited by background subtraction using the rubber band method (software OPUS8.1; Bruker Corporation, USA).

Dynamic light scattering (DLS) and zeta potential measurements were conducted using a Zetasizer Ultra device (Malvern Pananalytical, United Kingdom). For the analysis, 1 g L^−1^ of particle solutions was pipetted into the respective cuvettes for DLS (cuvettes PS macro; VWR, USA) and zeta (flow cell DTS1070; Malvern Panalytical, United Kingdom). The measurements were done using parameters for magnetite predefined in the corresponding software ZS Xplorer (Malvern Panalytical, United Kingdom).

Superconducting quantum interference device (SQUID) measurements with the magnetometer MPMS XL-7 (Quantum Design, Germany) were used to characterize magnetization. Around 10 mg of each particle sample was glued onto a small plastic tube (Fixogum; Marabu, Germany) before the analysis in a varying magnetic field (+/- 50 kOe) at 300 K.

Brunauer-Emmett-Teller (BET) surface areas were determined from nitrogen sorption isotherms at 77 K using an Autosorb iQ2 device (Anton Paar, Austria). In preparation, 100 mg of freeze-dried particle sample was outgassed for 5 h at 120 °C.

### 2.3 Ligand production

The plasmid containing the genetic information for the tetrameric version of the cysteine (cys)-tagged Z_Ca_ ligand (∼ 30 kDa) [22] was provided by the Department of Protein Science at KTH Royal Institute of Technology in Sweden. The Z_Ca_-cys ligand was expressed intracellularly in transformed *E. coli BL21 (DE3)* cells. Afterward, the protein was purified using IgG Sepharose 6 Fast Flow affinity resin (Cytiva, USA) following the procedure by Scheffel et al. [22].

The additionally studied affinity ligands rSpA and rSpA-cys (∼ 55 kDa) consisted of eight polymerized B domains of Protein A without and with a fused cys tag. The expression was also done in *E. coli BL21 (DE3)* as previously reported for the (RH)_4_-tagged rSpA version [27], and the purification was done similarly to the Z_Ca_-cys ligand [22] but without CaCl_2_ in the equilibration buffer.

### 2.4 Ligand immobilization onto magnetic nanoparticles

Depending on the experiment, ligand immobilization was done at 1 or 2 g *L*^−1^ particles and volumes of 0.3 to 1.0 mL (Protein LoBind Tubes; Eppendorf, Germany). The Z_Ca_-cys concentration per g particle varied in ligand adsorption isotherm experiments (0–1.5 g g^−1^) and was set to 0.3 g g^−1^ for all further studies. Immobilization procedures differed between the particle types. For bare MNP, the Z_Ca_-cys ligand was incubated with the particles for 1 h (1000 rpm, 25 °C; ThermoMixer C; Eppendorf, Germany) in 20 mM Tris at pH 7.0. Similarly, the immobilization onto MNP@TEOS particles was done in 20 mM NaH_2_PO_4_ at pH 6.0. The ligand immobilization onto MNP@GPTMS was done for 20 h (1000 rpm, 25 °C) in 1.4 M NaH_2_PO_4_ at pH 7.5. Following ligand immobilization, all particles were washed twice in the corresponding immobilization buffer by exchanging 95vol% of the supernatant with fresh buffer after magnetically separating the particles. In addition to washing with the immobilization buffer, the MNP@Z_Ca_-cys and MNP@TEOS@Z_Ca_-cys particles were incubated in all the applied process buffers (30 min, 1000 rpm, 25 °C) to remove not stably bound ligands. The MNP@GPTMS@Z_Ca_-cys particles were additionally blocked with 1 M Tris (pH 9) as done by Padwal et al. [40] and washed twice with 2 M NaCl, then 20 mM NaH_2_PO_4_, 500 mM NaCl (pH 7.4), and 20 mM sodium acetate, 500 mM NaCl (pH 4) to remove non-covalently attached ligands.

The same immobilization protocols were followed in studies with the engineered ligand variants rSpA and rSpA-cys employed to investigate the cys-specificity of the immobilization.

### 2.5 IgG binding and elution procedure

The interaction of the synthesized particles with purified Trastuzumab (IgG1) was analyzed. The general IgG binding and elution protocols were derived from the chromatography application of the calcium-dependent ligand published earlier [[19], [20], [21], [22]]. IgG binding was conducted in 50 mM Tris, 150 mM NaCl, 10 mM CaCl_2_ at pH 7.5 (TBSC). Throughout the studies, the particles were incubated with varying IgG concentrations (0–1.5 g g^−1^) under shaking at 1000 rpm and 25 °C. A binding duration of 1 h was chosen to ensure the equilibrium was reached under all experimental conditions (undersaturated and oversaturated). After IgG binding, the particles were magnetically separated and washed twice with TBSC by exchanging 95vol % of the particle supernatant. A third wash step was done with 5 mM NH_4_Ac, 5 mM CaCl_2_ (pH 5.5). For elution, the particles with adsorbed IgG were agitated in 100 mM ethylenediaminetetraacetic acid (EDTA) twice for 30 min (1000 rpm, 25 °C). In particle reuse studies, particles were cleaned with 0.3 M acetic acid at pH 3.3 after each use and equilibrated in TBSC before renewed IgG binding.

IgG recoveries were calculated by dividing the eluted IgG amount by the bound amount.

### 2.6 Protein analytics

Ligand concentrations in solution were determined using a bicinchoninic acid (BCA) assay (Pierce; Thermo Fisher Scientific, USA) following the instructions given by the manufacturer. If not stated differently, ligand loadings and adsorbed IgG amounts on particles were determined using a modified on-particle BCA assay described in [27]. As calibration standards, commercially available recombinant Protein A (Sino Biological Europe GmbH, Germany) or respectively pure IgG was used in the BCA assays. To evaluate the on-particle BCA assay, particle blanks of the same concentration as the particle sample were used to set the zero value.

Concentrations of pure IgG were determined photometrically with a NanoPhotometer (Implen, Germany) using the preinstalled parameters for human IgG.

A Bradford assay (Thermo Fisher Scientific, USA) was conducted to quantify EDTA-containing IgG samples (eluates). The manufacturer's user guide was followed and an IgG standard in the respective buffer was used.

Sodium dodecyl sulfate-polyacrylamide gel electrophoresis (SDS-PAGE) was applied to assess molecular weights and protein profiles in samples. A 5 % polyacrylamide stacking and 12 % running gel was employed. Samples were analyzed under reduced conditions (0.1 M dithiothreitol) and denatured before the analysis (95 °C for 5 min). Loaded gels were run in a Tris-Glycine buffer (AppliChem, Germany) at 110 mA, 180 V, 30 W (Invitrogen mini gel tank; Thermo Fisher Scientific, USA).

## 3 Results and discussion

### 3.1 Nanoparticle characterization

To ensure the successful synthesis of the three nanoparticle types we used for the subsequent Z_Ca_-cys immobilization, we analyzed the particles regarding their appearance and surface area (TEM, BET) and their functional groups (FT-IR). Furthermore, we determined their agglomeration behavior (DLS, zeta potential) and magnetization (SQUID) as relevant process parameters for magnetic separation.

**Fig. 2:**
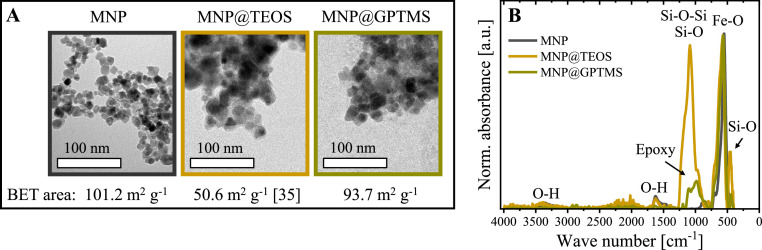
(**A**) TEM images and specific surface areas of the three particle types determined by BET nitrogen sorption isotherms. (**B**) FT-IR spectra of the particles. For better comparability, the spectra were normalized to their respective absorbance at ∼ 580 cm^−1^, representing Fe-O vibrations of iron oxide [39]. MNP: magnetic nanoparticle, TEOS: tetraethyl orthosilicate, GPTMS: (3-glycidyloxypropyl)trimethoxysilane.

TEM images of the bare MNP indicated a spherical to cubical shape with a mean particle size of 8.64 +/- 1.20 nm (Fig. 2.A), which agrees with literature values [41,42]. The TEOS and GPTMS coatings visually increased the particle sizes. With the larger particle sizes, the specific surface areas determined by BET sorption isotherms decreased from 101.2 m^2^ g^−1^ (MNP) to 93.7 m^2^ g^−1^ (MNP@GPTMS) and 50.6 m^2^ g^−1^ [35] (MNP@TEOS). This was expected because the specific surface area of coated particles is reduced as the surface-to-volume ratio is smaller for larger particles [35,43].

Based on the appearance and size analysis, the existence of coatings on MNP@TEOS and MNP@GPTMS in contrast to MNP was proven. With FT-IR spectroscopy, we further analyzed the functional groups on the particle surfaces (Fig. 2.B). As the coated particles were synthesized from bare MNP, we normalized all spectra to the peak at ∼580 cm^−1^, which can be attributed to Fe-O vibrations of iron oxides [39]. The coated particles showed additional absorption bands to the bare MNP, indicating successful particle modifications. The most prominent additional band of MNP@TEOS with a peak maximum at around 1050–1100 cm^−1^ is characteristic of Si-O-Si and Si-O stretching vibrations [39,[44], [45], [46]]. Furthermore, Si-O rocking vibrations (∼450 cm^−1^) and Si-O bending vibrations (∼ 805 cm^−1^) indicated the successful TEOS coating [44]. The absorbance peak at around 1630 cm^−1^ and the broad adsorption band at around 3300–3500 cm^−1^ probably represented O—H bonds that are characteristic functional groups of MNP and MNP@TEOS and were reported in the literature before [40,47,48]. MNP@GPTMS revealed a broad absorption peak at around 900–1100 cm^−1^ in FT-IR analytics, which can be assigned to Si-O and epoxide group stretching vibrations [39]. Further slight peaks manifesting a successful GPTMS coating were detected at around 2853 cm^−1^ and 2923 cm^−1^, representing alkyl groups [39,49]. From the FT-IR measurements, we concluded the successful surface modification and, thus, synthesis of the three particle types.

**Fig. 3:**
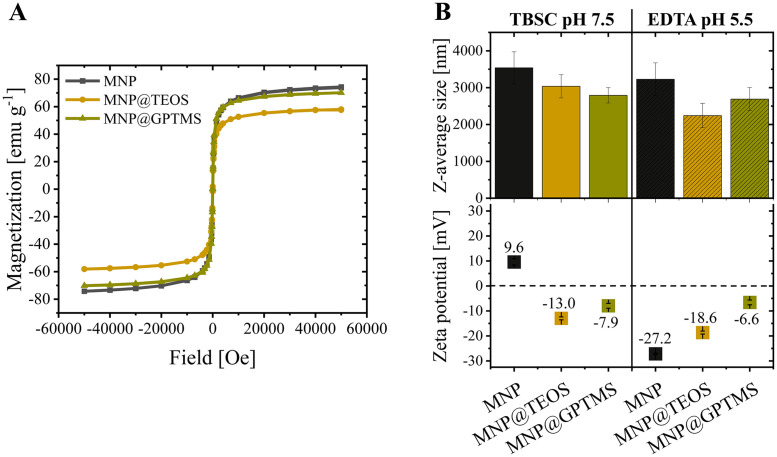
(**A**) Particle magnetizations determined by SQUID. (**B**) Hydrodynamic diameters of the particles as z-average values and zeta potential measurements in the exemplary process buffers TBSC (50 mM Tris, 150 mM NaCl, 10 mM CaCl_2_, pH 7.5) and EDTA (100 mM, pH 5.5). MNP: magnetic nanoparticle, TEOS: tetraethyl orthosilicate, GPTMS: (3-glycidyloxypropyl)trimethoxysilane.

Furthermore, we evaluated the particles' magnetic properties, which impact the capture duration in magnetic separation processes. SQUID measurements revealed the highest saturation magnetization for MNP (74 emu g^−1^), followed by MNP@GPTMS (70 emu g^−1^) and MNP@TEOS (58 emu g^−1^) (Fig. 3.A). The saturation magnetization of the bare MNP was in the expected range for iron oxide nanoparticles [33,35]. A reduction in the mass-specific saturation magnetization of the coated particles makes sense because of the additional mass of the coatings. The higher saturation magnetization of MNP@GPTMS compared to MNP@TEOS can probably be traced back to the thinner GPTMS coating, which can be assumed from the data in Fig. 1.A. Literature furthermore indicates that coatings may interact with atoms of the iron oxide core and form a magnetically disordered layer that reduces the total magnetic phase [50].

In addition, we performed DLS and zeta potential measurements to investigate the colloidal behavior and surface charge of the three particle types in the applied process buffers. Hydrodynamic diameters of the particles determined by DLS measurements ranged between 2250 and 3500 nm in TBSC (pH 7.5) and 100 mM EDTA (pH 5.5) (Fig. 3.B). As expected, the hydrodynamic diameters were larger than the particle core sizes seen in the TEM images (Fig. 2.A) because magnetic nanoparticles tend to agglomerate in solution due to electrostatic effects [51,52]. Nevertheless, literature indicates that the surface accessibility for proteins is still high despite the nanoparticle agglomeration [28].

The zeta potential of MNP shifted from positive in TBSC (9.6 +/- 1.2 mV) to negative in EDTA buffer (- 27.2 +/−0.3 mV), whereas the coated particles each showed similar zeta potentials in TBSC and EDTA (Fig. 3.B). The strong zeta potential shift observed for MNP indicated the binding of negatively charged EDTA molecules to the bare iron oxide surface. Our assumption agrees with the literature, where the binding of carboxyl groups of EDTA to iron oxides is widely reported [[53], [54], [55], [56], [57], [58]]. Furthermore, potential colloidal particle stabilization due to EDTA binding is described in literature [56], which is also consistent with the tendentially smaller mean hydrodynamic diameter of MNP in EDTA compared to TBSC (Fig. 3.B).

In addition to the EDTA interaction with bare MNP, the zeta potential measurements could also indicate potential interactions between EDTA and the coated particles. In both investigated process buffers, the MNP@GPTMS particles were similarly charged, and the MNP@TEOS particles had a slightly more negative net charge in EDTA at pH 5.5 compared to TBSC at pH 7.5, although higher zeta potentials were expected at pH 5.5.

**Fig. 4:**
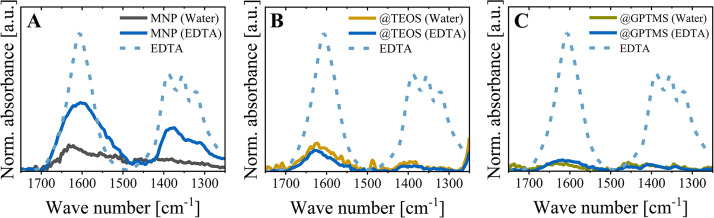
FT-IR spectra for the investigation of EDTA binding to (**A**) MNP, (**B**) MNP@TEOS and (**C**) MNP@GPTMS. Spectra were normalized to the absorbance at around 580 cm^−1^, assigned to Fe-O in the iron oxides [39]. Particles incubated in water serve as a reference (grey/orange/green lines). Particles incubated in 100 mM EDTA (pH 5.5, 1 h) were rebuffered into water by exchanging 95 vol% three times before the measurements (blue line). Pure EDTA (not normalized) is illustrated as a reference (blue dashed). MNP: magnetic nanoparticle, TEOS: tetraethyl orthosilicate, GPTMS: (3-glycidyloxypropyl)trimethoxysilane.

To further examine the EDTA interaction with the uncoated particles, FT-IR analytics of particles after incubation in EDTA (and rebuffering into water) were assessed as an orthogonal method (Fig. 4). With FT-IR spectra, EDTA binding to bare MNP surfaces was validated as particles showed absorption at around 1610 cm^−1^ and 1300-14,000 cm^−1^ (Fig. 4.A), which can be assigned to carboxyl groups in EDTA [58]. The peaks slightly shifted compared to pure EDTA due to its complex formation with the iron oxide surface. However, based on FT-IR measurements, EDTA adsorption onto MNP@TEOS and MNP@GPTMS particles could not be confirmed (Fig. 4.B and C). This hints at a stronger EDTA binding onto the uncoated MNP, which also agrees with the highest determined shift to negative zeta potential in EDTA buffer discussed before (Fig. 3.B). We concluded that the TEOS and GPTMS coatings reduced the interaction of the core iron oxide surface with EDTA.

In all our magnetic separation studies in this manuscript, the magnetic attraction of MNP@TEOS took longer than that of the other particles. With space-and-time-resolved extinction profiles, we quantified around twice the magnetophoretic velocities for MNP and MNP@GPTMS compared to MNP@TEOS in the process buffers TBSC and EDTA (Figure S.1). The different saturation magnetizations, chemical compositions and morphologies of the particles likely contributed to this finding. Based on the results, the synthesized MNP and MNP@GPTMS promise faster magnetic IgG separation processes than MNP@TEOS.

### 3.2 Z_Ca_ immobilization onto magnetic nanoparticles: capacities and mechanisms

**Fig. 5:**
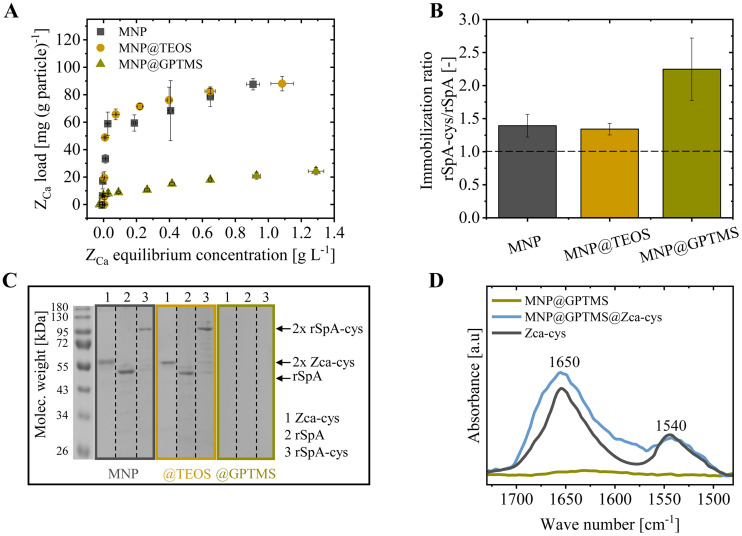
Investigation of ligand immobilization onto the three magnetic nanoparticle types. (**A**) Z_Ca_-cys ligand immobilization isotherms. (**B**) Ratio of rSpA-cys to untagged rSpA ligand immobilization. (**C**) Non-reducing SDS-PAGE analytics of particles with immobilized (1) Z_Ca_-cys, (2) rSpA and (3) rSpA-cys ligands (from different experiments). (**D**) FT-IR spectra of MNP@GPTMS, MNP@GPTMS@Z_Ca_-cys and pure Z_Ca_-cys in water. The spectra of particle samples were normalized to the absorbance at around 580 cm^−1^, assigned to Fe-O [39]. MNP: magnetic nanoparticle, TEOS: tetraethyl orthosilicate, GPTMS: (3-glycidyloxypropyl)trimethoxysilane.

Next, we examined the Z_Ca_-cys ligand immobilization onto the three synthesized magnetic particle types in various experiments (Fig. 5). First, we investigated ligand loadings in static adsorption isotherm experiments (Fig. 5.A). The maximum ligand adsorption onto 1 g of MNP (87.6 +/- 4.1 mg g^−1^) and MNP@TEOS (88.1 +/- 5.3 mg g^−1^) surpassed the adsorption onto MNP@GPTMS (24.3 +/- 2.7 mg g^−1^). Additionally, we investigated the impact of the cysteine tag on ligand immobilization by comparing the immobilized amount of a cys-tagged engineered Protein A (rSpA) with an untagged rSpA version (Fig. 5.B). The immobilization of the cys-tagged ligand version exceeded the untagged version for all three particles. Respective immobilization ratios were 1.39 +/- 0.17 for MNP, 1.34 +/- 0.09 for MNP@TEOS, and 2.25 +/- 0.47 for MNP@GPTMS (Fig. 5.B). Thereby, the rSpA-cys loadings on MNP, MNP@TEOS and MNP@GPTMS were with 78.3 +/- 5.0, 65.2 +/- 3.4 and 21.5 +/- 2.6 mg g^−1^ comparable to ZCa-cys. Furthermore, in our studies on Z_Ca_-cys immobilization, SDS-PAGE analytics of MNP@GPTMS with immobilized Z_Ca_-cys, rSpA, and rSpA-cys ligands revealed no protein bands (Fig. 5.C). In contrast, on ligand-immobilized MNP and MNP@TEOS, bands at around twice the molecular weight of the respective monomer were seen for Z_Ca_-cys (∼ 60 kDa) and rSpA-cys (∼ 110 kDa) and at the monomer weight for the untagged rSpA (∼55 kDa).

The stated results are first discussed for MNP and MNP@TEOS and then for MNP@GPTMS.

#### 3.2.1 MNP and MNP@TEOS

The maximum ligand loadings determined on MNP (87.6 mg g^−1^) and MNP@TEOS (88.1 mg g^−1^) are within the range of ligand loadings commonly reported in literature (50–210 mg g^−1^) [27,34,40,59]. However, comparing our ligand loadings with literature studies is difficult because we used a direct on-particle protein quantification method (Section 2.6) compared to the commonly done indirect back-calculation from unbound ligands. In our opinion, the quantification of ligand immobilization with on-particle protein quantification assays is more precise, as weakly/unspecifically bound ligands removed during the subsequent washing do not falsify the result.

Physical protein adsorption to bare MNP can generally occur via complex interactions, including electrostatic interactions, hydrogen bonds, hydrophobic interactions, and coordinative bonds [31]. Hydroxyl groups are the main interaction sites here [40]. Also, silica surfaces, as present in MNP@TEOS, are known to interact physically with various proteins over a wide pH range [60]. The observed superior rSpA-cys adsorption onto MNP and MNP@TEOS compared to untagged rSpA (Fig. 5.B) indicates a beneficial impact of the cysteine tag on the physical Z_Ca_-cys immobilization. We assume that cys-tagged ligands formed homodimers that promoted the physical immobilization stability due to a larger contact area with the particle surface than monomeric proteins, which can add up to a generally stronger interaction [61]. This explanation seems reasonable, given our observation of the immobilized dimeric ligand forms of the cys-tagged Z_Ca_ and rSpA ligands on MNP seen in non-reducing SDS-PAGE analytics (Fig. 5.C). Likely, the cysteines oxidized to cystine under S-S bond formation upon reduction of Fe^3+^ ions on the iron oxide surface, as reported before [62,63]. The oxidation of thiol-containing molecules to disulfides by silica surfaces has also been reported in a recent publication [64] and agrees with the Z_Ca_-cys and rSpA-cys homodimers seen on MNP@TEOS in SDS-PAGE. To our knowledge, the possibility of immobilizing cysteine-tagged molecules onto particle surfaces via dimer formation has not been reported before.

In comparable immobilization studies with an untagged Protein G ligand, Padwal et al. reached higher physical ligand binding to 1 g of MNP than to silica-coated particles, which they explained with the larger specific surface area of the bare particles [40]. In contrast, we observed similar Z_Ca_-cys adsorption onto MNP (87.6 mg *g*^−1^) and the silica-coated MNP@TEOS (88.1 mg g^−1^), although the bare MNP have a larger specific surface area (Fig. 2.A). This points at more efficient ligand binding to the MNP@TEOS than to the MNP surface and could thus indicate beneficial interactions of the Z_Ca_-cys molecules with the silica. We washed both particle types in EDTA buffer during immobilization (Section 2.4). Hence, the enhanced EDTA binding to the MNP surface discussed in Section 3.1 could have negatively impacted the physical ligand adsorption. In general, competitive ligand displacement by EDTA or electrostatic repulsion between the negatively charged Z_Ca_-cys ligands (theoretical isoelectric point of 4.77 [65]) and the highly negatively charged MNP surface in the EDTA buffer (Fig. 3.B) could have occurred.

#### 3.2.2 MNP@GPTMS

The MNP@GPTMS with intended covalent ligand immobilization were washed with buffers of high ionic strength and different pH values between 4 and 7 after the ligand immobilization (Section 2.4). These washing steps successfully removed physically bound ligand molecules from the surfaces, as we concluded from missing ligand bands in SDS-PAGE analytics of the ligand-immobilized particles (Fig. 5.C). Unlike physically attached ligands on MNP and MNP@TEOS, covalently bound ligands were likely still attached to the particles remaining in the gel loading pocket during the electrophoresis and can thus not be seen on the separating gel. However, the covalently bound ligand molecules, not seen in SDS-PAGE analytics, were quantitatively confirmed by the on-particle BCA protein quantification assay performed to analyze the Z_Ca_-cys isotherm loadings (Fig. 5.A). Furthermore, in FT-IR analytics of MNP@GPTMS@Z_Ca_-cys performed as a qualitative orthogonal method (Fig. 5.D), bands at 1650 cm^−1^ and 1540 cm^−1^ were detected and assigned to C = O stretching, N—H bending, and C—N stretching vibrations in the ligand [66].

In general, proteins can covalently bind to epoxy groups via various nucleophilic groups present in amino acids (such as amino and thiol groups) that cause the epoxy ring opening. However, we detected a two to three-times higher immobilization of rSpA-cys compared to the untagged version (Fig. 4.B), which indicated a preferred and effective interaction of the epoxy with the thiol of the cysteine. Thiol groups in cysteine side chains may have a higher nucleophilic reactivity at the neutral pH used during immobilization [67,68], probably causing the observed reaction specificity. As cysteine is only present in the tag of rSpA-cys and Z_Ca_-cys [19,27], the cys-mediated, site-directed immobilization onto MNP@GPTMS was likely enhanced. We think it is rather unlikely that the increased binding of cys-tagged ligands seen in Fig. 4.B is explained by the immobilization of formed dimers, as the effect is more significant than observed for MNP and MNP@TEOS.

The number of epoxy binding sites was presumably less than the many physical interaction possibilities discussed above for MNP and MNP@TEOS, which likely contributed to the lower determined Z_Ca_-cys loadings (Fig. 5.A). Nevertheless, the maximum detected Z_Ca_-cys loading on MNP@GPTMS (24.3 mg g^−1^) agrees with expectations based on literature. In a study on bovine serum albumin (BSA) immobilization onto epoxy silane nanoparticles, Zhang et al. reported a BSA loading of 25 mg g^−1^ after removing weakly bound proteins, which is similar to our Z_Ca_-cys immobilization (24.3 mg g^−1^) [69] (although BSA is a larger protein than Z_Ca_-cys, meaning more ligand molecules were probably bound in our study).

From the discussed results in Section 3.2, we concluded the successful immobilization of Z_Ca_-cys onto the three synthesized particle types - physically and non-oriented mainly through dimer formation on MNP and MNP@TEOS, while covalently and more site-directed on MNP@GPTMS.

### 3.3 IgG Interaction with magnetic nanoparticles@Z_C__a_-cys

#### 3.3.1 IgG binding capacities

**Fig. 6:**
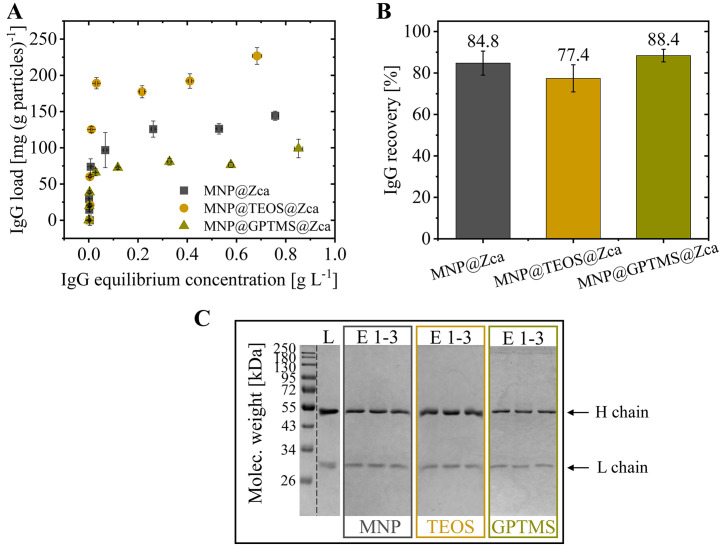
IgG adsorption and desorption onto/from magnetic nanoparticles@Z_Ca_. (**A**) Adsorption isotherms onto particles in TBSC buffer (50 mM Tris, 150 mM NaCl, 10 mM CaCl_2_, pH 7.5). (**B**) IgG recoveries after incubation of 110 mg IgG per g particles, washing, and elution in 100 mM EDTA (pH 5.5). The bound IgG was calculated from the initially loaded and unbound IgG. (**C**) Reduced SDS-PAGE of loaded IgG (L) and elution samples taken in three subsequent particle reuse cycles (E 1–3) of the three functionalized particles. The heavy (H; ∼ 50 kDa) and light (L; ∼ 25 kDa) chains of the IgG can be seen. MNP: magnetic nanoparticle, TEOS: tetraethyl orthosilicate, GPTMS: (3-glycidyloxypropyl)trimethoxysilane.

The IgG binding capacities onto the magnetic particles were investigated in adsorption isotherm studies. For this, the particles were immobilized with initial 0.3 g Z_Ca_-cys per g particles, which led to ligand loadings of 73.5 +/- 5.0 mg g^−1^ (MNP@Z_Ca_-cys), 76.1 +/- 6.9 mg g^−1^ (MNP@TEOS@Z_Ca_-cys) and 22.7 +/- 1.7 mg g^−1^ (MNP@GPTMS@Z_Ca_-cys). The conducted isotherms revealed the highest IgG binding onto MNP@TEOS@Z_Ca_-cys particles (226.7 +/- 11.4 mg g^−1^), followed by MNP@Z_Ca_-cys (144.3 +/- 6.3 mg g^−1^) and MNP@GPTMS@Z_Ca_-cys (108.3 +/- 1.9 mg g^−1^) (Fig. 6.A). However, the isotherms of all particles showed an increasing IgG immobilization after a plateau phase, indicating an IgG double-layer formation due to IgG-IgG interactions at high antibody concentrations. To ensure specific ligand-IgG interactions, the monolayer coverage should not be surpassed. For evaluation of the monolayer formation, the first seven data points of each isotherm were fitted with a Langmuir fit (Supporting Information, Section S.2). Thereby, coefficients of determination (R^2^) were 0.99, indicating a suitable fit. With the Langmuir fit, maximum monolayer IgG loadings of 196 mg g^−1^ (MNP@TEOS@Z_Ca_-cys), 128 mg g^−1^ (MNP@Z_Ca_-cys), and 78 mg g^−1^ (MNP@GPTMS@Z_Ca_-cys) were estimated. Furthermore, the equilibrium constant $K_{L}$, an indicator of the binding affinity, was calculated to be 126 L g^−1^ for MNP, 78 L g^−1^ for MNP@TEOS, and 322 L g^−1^ for MNP@GPTMS (Table S.1).

Although the MNP@Z_Ca_-cys and MNP@TEOS@Z_Ca_-cys had similar quantities of bound ligand molecules per g particle in this experiment (73.5 +/- 5.0 and 76.1 +/- 6.9 mg g^−1^), the maximum IgG binding on the MNP@TEOS@Z_Ca_-cys was about 70 mg g^−1^ higher. Thus, more effective IgG binding to MNP@TEOS@Z_Ca_-cys was concluded. One explanation for this finding could be the beneficial electrostatics between MNP@TEOS and IgG molecules. As seen before, the MNP@TEOS particles have a negative net surface charge in TBSC binding buffer (pH 7.5), whereas the MNP have a positive net charge (Fig. 3.B). Thus, IgG molecules with a positive net charge (IEP: 8.7–9.1 [70]) probably got more electrostatically repelled by the bare MNP than the TEOS-coated surface, which could have hampered the IgG adsorption. Supporting this explanation, Yang et al. reported that electrostatic repulsion between IgG and adsorbent surfaces can negatively impact the IgG binding, especially in the case of non-oriented ligand binding sites that have a close distance to the surface, as assumed for our MNP@Z_Ca_-cys [71].

The IgG adsorption onto 1 g of MNP@GPTMS@Z_Ca_-cys was lower than onto MNP@Z_Ca_-cys and MNP@TEOS@Z_Ca_-cys (Fig. 6.A). The lower ligand load per g MNP@GPTMS discussed in Section 3.2 resulted in fewer IgG binding sites, which likely contributed to this observation. However, while the ligand load on MNP and MNP@TEOS was around 3.7 times larger than on MNP@GPTMS, the IgG binding differed by factors of 1.6 and 2.5. This indicates a higher IgG binding ratio to the ligands and, thus, more efficient IgG binding onto MNP@GPTMS@Z_Ca_-cys. More efficient IgG binding to the ligand is also implied by the much higher $K_{L}$ value estimated in the Langmuir fit (Table S.1). A plausible explanation for the finding is the enhanced ligand orientation on MNP@GPTMS@Z_Ca_-cys compared to the other particles (Section 3.2). It is generally known that site-directed immobilization of Protein A-based ligands benefits the subsequent IgG binding through better accessibility [59,71,72].

We further want to mention that the varying ligand densities on the three particle types used in this experiment could also have impacted the IgG binding [27,40,73]. Ligands provide binding sites for target molecules, but steric hindrance can arise when loading densities get too high on the particle surface. The highest specific ligand density per area of the investigated particles was estimated for the TEOS-coated particles (Figure S.2). Thus, the higher IgG binding efficiency onto MNP@TEOS@Z_Ca_-cys particles compared to MNP@Z_Ca_-cys did not reveal steric hindrance effects. In general, the Z_Ca_-cys density on MNP@GPTMS particles was lower than on the other particle types, which could have contributed to the better accessibility of the ligand binding sites. However, the investigation of steric effects due to different ligand densities was not the focus of our study.

As our ligand adsorption studies, our IgG adsorption studies cannot be directly compared to literature values because we used an on-particle protein quantification assay in contrast to the recalculation from the unbound IgG usually done in literature. With the on-particle protein quantification, we expected to determine lower IgG loadings due to washed-off, unspecifically/weakly bound IgG molecules that would usually be detected as bound. However, several literature studies on Protein A immobilized magnetic particles reported similar or even lower IgG capacities between 50 and 100 mg g^−1^ [59,74,75]. Kim et al. site-directedly immobilized cysteine-tagged Protein A ligands onto magnetic nanoparticles via a two-step chlorophenylsilane modification and reported a ligand load of 72–80 mg g^−1^ and an IgG binding capacity of around 100 mg g^−1^ [59]. Although they worked with a different Protein A ligand, a comparison with our study indicates a more efficient IgG-to-ligand binding ratio for all our particles. Furthermore, Iype et al. bound 4–5 g g^−1^ Protein A onto bare iron oxide nanoparticles by cross-linking and separated 100–160 mg IgG per g particle [75], which depicts a noticeably less efficient ligand usage than we achieved with all our investigated particles.

#### 3.3.2 IgG desorption: Z_Ca_ functionalities and immobilization stabilities

Recovery experiments in 100 mM EDTA at pH 5.5 revealed the highest desorption efficiency from MNP@GPTMS@Z_Ca_-cys (88.4 +/- 3.0 %), followed by MNP@Z_Ca_-cys (84.8 +/- 5.8 %) and MNP@TEOS@Z_Ca_-cys (77.4 +/- 6.6 %) (Fig. 6.B). The IgG recovery from MNP@GPTMS@Z_Ca_-cys is in the upper range of already reported magnetic particle studies [[76], [77], [78]]. As for IgG adsorption, the enhanced site-directed ligand immobilization mediated by the epoxy groups of MNP@GPTMS probably contributed to the highest detected desorption efficiency. It is plausible that the conformational change of the ligand required for IgG desorption upon Ca^2+^ removal [19] is more efficient if the ligand is directed and not randomly attached to the particle surface. Furthermore, the general ligand accessibility is improved, which could have simplified the IgG desorption similar to the adsorption.

The mean recoveries from MNP@Z_Ca_-cys and MNP@TEOS@Z_Ca_-cys were only 4 to 11 % lower than those from MNP@GPTMS@Z_Ca_-cys. This also indicates high calcium-dependent ligand functionalities. Effective conformational changes of the mainly as homodimer immobilized ligands upon calcium depletion were thus possible. However, we assume that unspecifically adsorbed IgG slightly falsified the result for the MNP@Z_Ca_-cys particles. By analyzing the IgG interaction with blank MNP, we observed that a small amount of IgG molecules unspecifically adsorbed onto the bare MNP surface and desorbed in the EDTA elution buffer (9.5 +/- 3.7 mg g^−1^ by Bradford assay). In contrast, no IgG was detected in elution fractions of blank MNP@TEOS and MNP@GPTMS particles. We concluded that the measured IgG elution from the MNP@Z_Ca_-cys was likely distorted by unspecific binding, and the real, ligand-mediated IgG recovery was probably slightly lower.

In a control study with the pH-sensitive rSpA-cys ligand, we observed similar IgG binding onto the three investigated particles@rSpA-cys as to the particles@Z_Ca_-cys in TBSC buffer (pH 7.5). However, significantly lower IgG recoveries were reached in 100 mM EDTA (pH 5.5) with a maximum of 5 % from the MNP@rSpA-cys particles (Figure S.3). The findings make sense because IgG binds to Protein A-based ligands at neutral pH, but the pH of 5.5 was not acidic enough to promote efficient IgG desorption from the pH-dependent rSpA-cys ligands. The nevertheless measured low recovery can probably be explained by unspecific IgG interaction and was among the particles, thus the highest for the MNP@rSpA-cys.

Lastly, it is notable that high ligand immobilization stabilities were observed. The particles with immobilized Z_Ca_-cys can be reused in (minimum) three consecutive cycles of IgG adsorption, washing, and elution steps, as revealed by consistent IgG elution (Fig. 6.C, lanes E 1–3). Furthermore, no ligand leaching into the eluted IgG fractions was detected in SDS-PAGE. Both observations indicate stable ligand immobilization on the particles and validate the removal of not-stably bound ligands during the immobilization procedure (Section 2.4).

## 4 Conclusion and outlook

By combining the calcium-dependent affinity ligand Z_Ca_-cys with the magnetic separation technique, we aim to enhance current monoclonal antibody (mAb) downstream processing (DSP). As a first step towards calcium-dependent magnetic separation processes, we investigated physical and covalent immobilization strategies of the cysteine-tagged Z_Ca_ ligand onto three magnetic nanoparticle types: bare iron oxide magnetic nanoparticles (MNP) and MNP coated with tetraethyl orthosilicate (MNP@TEOS) and (3-glycidyloxypropyl)trimethoxysilane (MNP@GPTMS). Oxidized Z_Ca_-cys ligand homodimers physically (probably randomly) adsorbed to MNP and MNP@TEOS particles. The main immobilization mechanism onto MNP@GPTMS differed from the other particles as a cysteine tag-promoted epoxy ring opening reaction realized covalent Z_Ca_-cys immobilization with enhanced ligand orientation. In our studies, the MNP@Z_Ca_-cys particles emerged as the least suitable particles, mainly due to (1) EDTA interactions with the bare iron oxide particles hampering efficient calcium-dependent IgG binding and (2) unspecific IgG interaction with the bare particle surface. In contrast, our results show that the two coated particles (MNP@TEOS and MN@GPTMS) have suitable characteristics for calcium-dependent magnetic separation. Both particle types are simple to synthesize in one-step MNP modifications, which is practical for future scale-ups. Comparing the two coated particle approaches, the MNP@TEOS@Z_Ca_-cys particles stand out due to the highest reached IgG binding (196 mg g^−1^). This is explainable by large ligand loadings (88 mg g^−1^), which is possible with physical immobilization. However, the MNP@GPTMS@Z_Ca_-cys particles that rely on covalent ligand immobilization are also highly suitable for the intended process. Firstly, due to the enhanced ligand orientation, IgG bound very efficiently to the (costly) ligand molecules. Secondly, the IgG recovery from MNP@GPTMS@Z_Ca_-cys (88 %) was in the upper range of reported literature values and exceeded the MNP@TEOS@Z_Ca_-cys particles by 11 %. Thirdly, the determined high saturation magnetization of MNP@GPTMS (70 emu g^−1^) and the almost double as fast measured magnetophoretic velocity of the GPTMS- compared to the TEOS-coated particles in process buffers enables faster magnetic capture, which is beneficial for high process productivity. Fourthly, the material costs for the MNP@GPTMS synthesis are nearly five times cheaper than those of MNP@TEOS.

The designed particles pave the way for the subsequent development of calcium-dependent magnetic mAb separation processes. For further process development, we plan to screen an alternative elution buffer composition to EDTA in order to implement an economical and sustainable process. Sodium chloride or citrate are promising candidates, as efficient IgG elution from Z_Ca_-cys has already been accomplished in chromatography studies. Furthermore, we want to investigate the capture of antibodies directly from crude cell culture, which is a distinct advantage of magnetic separation enabled by the general non-porosity of the particle adsorbent (clogging is prevented). The direct capture of mAbs circumvents the traditional requirement for clarification steps, reducing process time and costs. Thus, the approach is highly promising for process intensification. Also, we plan to optimize step durations (e.g., for mAb capture and elution) and scale up the process into an automated pilot scale plant. Lastly, it would also be interesting to investigate the performance of ligand constructs with a higher Z_Ca_ domain number. In chromatography, steric hindrance effects due to ligand polymerization are one of the main limitations of optimizing binding capacities, which might be possible to overcome with the non-porosity of the magnetic particle adsorbents.

The calcium-dependent magnetic mAb separation approach is still in its infancy, but its potential benefits for mAb DSP are promising.

## CRediT authorship contribution statement

**Ines Zimmermann:** Writing – original draft, Visualization, Validation, Methodology, Investigation, Formal analysis, Data curation, Conceptualization. **Friederike Eilts:** Writing – review & editing, Supervision, Conceptualization. **Anna-Sophia Galler:** Validation, Investigation, Formal analysis. **Jonas Bayer:** Validation, Investigation. **Sophia Hober:** Writing – review & editing, Resources, Conceptualization. **Sonja Berensmeier:** Writing – review & editing, Supervision, Resources, Project administration, Funding acquisition, Conceptualization.

## Declaration of competing interest

The authors declare the following financial interests/personal relationships which may be considered as potential competing interests:

SH holds a patent regarding utilization of the ZCa domain. If there are other authors, they declare that they have no known competing financial interests or personal relationships that could have appeared to influence the work reported in this paper.

## Data Availability

Data will be made available on request.

## Appendix Supplementary materials

Image, application 1
