# Supplementary material for: Immobilizing calcium-dependent affinity ligand onto iron oxide nanoparticles for mild magnetic mAb separation
Source: Biotechnol Rep (Amst). 2024 Nov 26;45:e00864. doi: 10.1016/j.btre.2024.e00864 (PMC11647653; doi:10.1016/j.btre.2024.e00864)
Supplement: Supplementary file 1 [file mmc1.pdf]

Supplementary Material for

Immobilizing Calcium-Dependent Affinity Ligand onto Iron Oxide Nanoparticles for Mild Magnetic mAb Separation

Ines Zimmermann [a], Friederike Eilts [a], Anna-Sophia Galler [a], Jonas Bayer [b], Sophia Hober [c], Sonja Berensmeier *, [a, b]

[a] Chair of Bioseparation Engineering, TUM School of Engineering and Design, Technical University of Munich, Boltzmannstraße 15, 85748 Garching, Germany

[b] Munich Institute of Integrated Materials, Energy and Process Engineering, Technical University of Munich, Lichtenbergstraße 4a, 85748 Garching, Germany

[c] Department of Protein Science, KTH Royal Institute of Technology, SE-106 91 Stockholm, Sweden

E-mail:
Sonja Berensmeier (*corresponding author): s.berensmeier@tum.de

Keywords: anything but conventional chromatography (ABC), downstream processing, epoxy, silica, physical and covalent immobilization

S. 1 Additional results

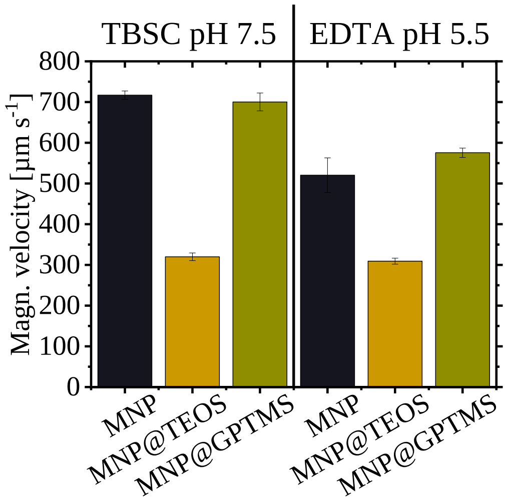

Figure S.1. Magnetophoretic sedimentation velocities of particles in TBSC buffer (50 mM Tris, 150 mM NaCl, 10 mM CaCl2, pH 7.5) and EDTA (100 mM, pH 5.5). Magnetophoretic velocities of the particles were determined using a LUMiReader (LUM GmbH, Germany) equipped with five stacked cylindrical neodymium boron ferrite (NdFeB) magnets (Webcraft GmbH, Germany). The magnetic field strength over the magnets can be found in [1]. Based on space-and-time-resolved extinction profiles at 870 nm, 630 nm, and 420 nm, the magnetophoretic velocities were determined using the software PSA-Wizard (SEPview software; LUM GmbH, Germany).

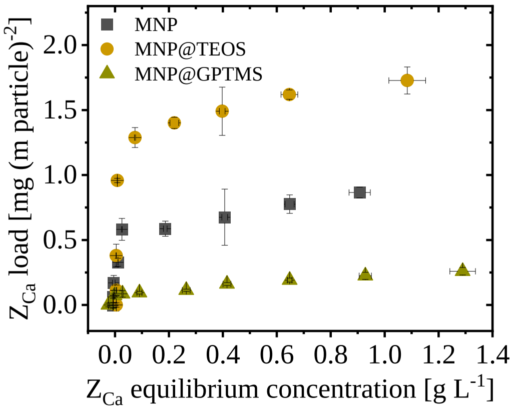

Figure S.2. Z_{Ca}-cys immobilization isotherms related to specific BET surface areas. The loadings shown in Figure 5.A were normalized to the specific surface areas determined by BET nitrogen sorption isotherms. It has to be noted that BET sorption isotherms are valid for an approximation of the ligand densities on the surfaces. However, detailed pore and accessibility analysis are needed for more precise statements.

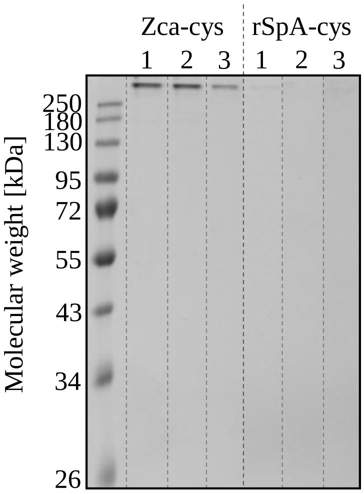

Figure S.3. Non-reduced SDS-PAGE of IgG eluates desorbed from (1) MNP, (2) MNP@TEOS and (3) MNP@GPTMS with immobilized Z_{Ca}-cys and rSpA-cys ligands in 100 mM EDTA at pH 5.5. IgG visually desorbed from the particles@Z_{Ca}-cys, whereas maximum 5% IgG recovery was determined from the MNP@rSpA. The antibody (~ 150 kDa) migrated in the gel higher than expected, probably due to its branched structure under the non-reduced conditions. MNP: magnetic nanoparticle, TEOS: tetraethyl orthosilicate, GPTMS: (3-glycidyloxypropyl)trimethoxysilane.

S. 2 Langmuir fit of IgG adsorption isotherms

The Langmuir isotherm model is given by equation (1) below [2].

$$q = \frac{q_m K_L C}{1 + K_L C} \qquad (1)$$

q	:	adsorbed IgG [mg g^{-1}]
K_L	:	equilibrium constant [L g^{-1}]
C	:	IgG concentration [g L^{-1}]
q_m	:	max. IgG loading [mg g^{-1}]

The Langmuir isotherm underlies the assumptions of monolayer binding and equal binding sites. The K_L value can be used as an affinity indicator, with higher values generally stating higher binding affinities. The Langmuir isotherm can be linearized to the form in equation (2), which simplifies the estimation of the Langmuir parameters q_m and K_L. By plotting $\frac{C}{q}$ over C, a linear form is obtained with an intercept of $\frac{1}{K_L q_m}$ and a slope of $\frac{1}{q_m}$.

$$\frac{C}{q} = \frac{1}{K_L q_m} + \frac{1}{q_m} C \qquad (2)$$

Figure S.4 shows the IgG adsorption isotherm data from Figure 6.A plotted in linearized form.

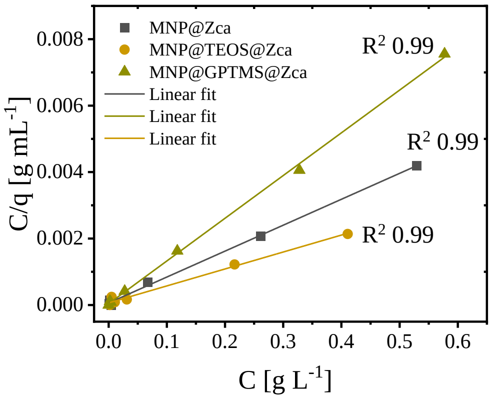

MNP@GPTMS@Zca $\frac{C}{q} = 4.0081 \cdot 10^{-5} + 0.0129 \cdot C$

MNP@Zca $\frac{C}{q} = 6.1986 \cdot 10^{-5} + 0.0078 \cdot C$

MNP@TEOS@Zca $\frac{C}{q} = 6.5211 \cdot 10^{-5} + 0.0051 \cdot C$

Figure S.4. Linearized Langmuir fit (based on equation 2) of the IgG adsorption isotherm data presented in Figure 6.A. The first seven isotherm data points were used. The coefficients of determination (R^2) of the fits are given, and the linear fit equations are next to the figure. Data was plotted and fitted in OriginPro 2021b.

From the slope and the intercept of the three linear fits, the values for q_m and K_L were estimated (Table S.1).

Table S.1 Langmuir parameters derived from the linear fits of the IgG adsorption isotherms (Figure S.4).

	q_m [mg g^{-1}]	K_L [L g^{-1}]
**MNP@Z$_{Ca}$**	128.2	125.8
**MNP@TEOS@Z$_{Ca}$**	196.1	78.2
**MNP@GPTMS@Z$_{Ca}$**	77.5	321.8

References

[1] I. Zimmermann, Y. Kaveh-Baghbaderani, F. Eilts, N. Kohn, P. Fraga-García, S. Berensmeier, Direct Affinity Ligand Immobilization onto Bare Iron Oxide Nanoparticles Enables Efficient Magnetic Separation of Antibodies, ACS Appl. Bio Mater. (2024). https://doi.org/10.1021/acsabm.4c00280.

[2] G. Carta, A. Jungbauer, Protein Chromatography, Wiley, 2020.
